# Supplementary material for: A One-Month Advanced Glycation End Products—Restricted Diet Improves CML, RAGE, Metabolic and Inflammatory Profile in Patients with End-Stage Renal Disease Undergoing Haemodialysis
Source: Int J Mol Sci. 2024 Aug 15;25(16):8893. doi: 10.3390/ijms25168893 (PMC11354996; doi:10.3390/ijms25168893)
Supplement: Supplementary file 1 [file ijms-25-08893-s001.zip › ijms-3078037-supplementary.pdf]

Supplementary Table S1. Haematological and several biochemical markers of the intervention and control group.

	Intervention Group (N=22) mean± standard deviation or median (25th, 75th percentile) (min, max)		p-value	Control Group (N=20) mean± standard deviation or median (25th, 75th percentile) (min, max)		p-value	% difference * (intervention)	% difference * (control)	p-value
	Baseline	Follow-up		Baseline	Follow-up				
Full Blood Count									
Red blood cells, RBC ($x10^3/mm^3$) †	3.86 (3.50, 4.12)	3.75 (3.40, 4.32)	0.543	3.65 (3.40, 3.97)	3.79 (3.40-4.12)	0.494	-0.51± 6.78	1.55±11.51	0.476
Mean corpuscular volume, MCV (fl)†	89 (86, 9 4)	89 (86, 94)	0.685	92 (88, 94)	92 (86-96)	0.741	-0.27± 2.80	0.07±2.43	0.671
Red cell distribution width, RDW-CV (%) †	15.8 (14.2, 16.0)	14.9 (14.9, 15.6)	**0.024**	15.0 (14.1, 16.0)	15.8 (14.6, 17.3)	0.158	-2.98± 4.87	13.79± 37.31	0.085
White Blood Cells, WBC ($x10^3/mm^3$)	7.5 (6.8, 9.3)	7.3 (6.3, 9.6)	0.587	7.8 (6.8, 9.8)	7.7 (6.4, 9.5)	0.809	-1.56± 22.00	-1.53± 22.24	0.997
Neutrophils (thousands/mm^3)	5.4 ± 2.0	6.0 ± 2.0	0.100	6.3 ± 1.8	5.4 ± 1.9	0.725	39.96 (-7.0, 3.73)	0.00 (-19.45, 14.16)	0.273
Lymphocytes (%)	1.4± 0.3	1.3 ± 0.3	0.163	1.5 ± 0.5	1.6 ± 0.4	0.745	-6.20 (-11.94, 6.47)	0.00 (-14.61, 17.58)	0.240
Haematocrit, Ht (%)	34.5 ± 3.5	34.6± 5.6	0.410	33.5 ± 3.3	33.1± 5.3	0.707	0.00 (-4.63, 4.77)	2.43(-4.14, 6.12)	0.450
Haemoglobin, Hb (g/dl)	11.3 ± 1.1	11.1 ± 1.3	0.371	10.8 ± 1.1	10.8 ± 1.2	1.000	-1.50±8.11	0.19±7.93	0.496
Mean corpuscular Hb, MCH (pg)	29.4 (28.1, 30.9)	28.9 (27.7, 30.9)	**0.005**	29.5 (27.9, 31.3)	29.30 (27.5, 30.6)	0.076	-1.67±3.48	-1.29±3.18	0.713
Mean corpuscular Hb concentration, MCHC (g/dL)	32.6 (32.1, 33.0)	31.8 (31.5, 32.6)	**0.001**	32.4 (31.3, 32.9)	31.6 (31.0, 32.4)	**0.004**	-1.66 (-2.73, -0.98)	-1.82 (-3.01, -0.15)	0.968
Platelets, PLT (thousands/mm^3)	209 ± 48	206 ± 49	0.660	213 ± 60	219.± 68	0.617	-0.70 (-6.40, 7.62)	-3.43 (-14.39, 17.54)	0.821
Mean platelet volume, MPV (μm^3) †	9.6 (9.0, 10.2)	9.8 (9.3, 10.3)	0.088	9.5 (8.9, 10.5)	9.5 (9.2, 10.1)	0.604	1.09 (-2.26, 5.22)	0.00 (-2.00, 2.27)	0.411
Electrolytes									
Sodium, Na^+ (mmol/L)	136 ± 2	136 ± 2	0.781	137 ± 3	138 ± 2	0.217	0.00(-0.92, 1.65)	0.00 (-1.25, 1.94)	0.497
Potassium, K^+ (mmol/L)	5.2 ± 0.5	5.6 ± 0.6	**0.025**	4.7 ± 0.8	5.1 ± 0.8	**0.024**	8.55±14.85	8.70±15.68	0.975
Calcium, Ca^{++} (mg/dl) †	9.2 (8.6, 9.6)	9.6 (9.2, 9.9)	**0.001**	9.4 (9.2, 9.6)	9.6 (9.3 , 9.9)	**0.002**	3.17 (1.12, 6.80)	3.15 (1.03, 4.30)	0.497
Phosphate, P (mmol/L) †	3.30 (3.90, 4.95)	4.10 (3.15, 4.77)	0.651	3.90 (2.82, 4.80)	3.50 (2.95, 6.37)	0.468	6.10±28.88	14.38±43.15	0.473
Enzymes									
Creatine kinase, CK (U/l)	59± 29	63± 32	0.922	41 ± 22	41± 21	0.635	-14.81 (-26.43, 6.61)	-16.59 (7.04, 21.39)	0.183

Alanine aminotransferase, ALT (U/L) †	13 (11, 16)	14 (12, 17)	0.963	16 (11, 18)	11 (11, 16)	0.066	0.00 (-15.96, 22.50)	-25.81 (-8.71, 0.00)	0.120
Aspartate aminotransferase, AST (U/L) †	15 (13, 18)	15 (13, 19)	0.578	16 (12, 20)	16 (12, 20)	0.463	0.00 (-9.12, 7.41)	-6.90 (-23.75, 7.91)	0.354
γ-glutamyl transferase, γGT (U/L) †	20 (12, 30)	19 (13, 31)	0.159	19 (14, 22)	18 (15, 21)	0.540	7.41 (-7.94, 18.63)	7.17 (-6.74, 14.94)	0.980
Alkaline phosphatase, ALP (U/L) †	87 (64 , 99)	84 (57, 106)	0.168	83 (60, 95)	79 (63, 92)	0.409	-3.16±13.14	-1.65±13.29	0.714
Bilirubin (mg/dl)	0.3 ± 0.1	0.3 ± 0.1	0.158	0.2 ± 0.1	0.2± 0.1	0.506	-9.82 (-23.71, 33.33)	-2.08 (-19.97, 12.97)	0.571

†The variables were transformed as follows to achieve normality and conduct parametric tests for comparisons.
Logarithmized variables (log variable) at baseline and follow-up: AST, CK, ALP, C, P
Reversed variables (1/variable) at baseline and follow-up: RBC, ALT, RDW, GGT.
Squared variables at baseline and follow-up: MCV, MPV.
*Baseline value significantly different between intervention and control group.
NA: Not applicable.

Supplementary Table S2: Changes in selected biochemicals parameters after 2 months**.**

	Intervention Group (N=22) mean± standard deviation or median (25th, 75th percentile)			p-value (0-2 months)	Control Group (N=20) mean± standard deviation or median (25th, 75th percentile)			p-value (0-2 months)	% difference * (intervention)	% difference * (control)	p-value	p-value (repeated measures ANOVA)
	Baseline	Follow-up (1 month)	Follow-up (2 months)		Baseline	Follow-up (1 month)	Follow-up (2 months)					
Uric acid (mg/dl)	6.6±1.3	6.8± 1.2	6.8± 1.1	0.238	6.1± 1.1	6.1±1.4	6.2±1.2	0.854	3.2± 9.6	1.3± 14.6	0.627	0.402
HbA1c (%)†	5.6 (5.1, 6.4)	5.4 (5.0, 6.1)	5.0 (4.7, 5.8)	**<0.001**	5.7 (5.3, 7.1)	5.7 (5.3, 7.4)	5.8 (5.4, 7.5)	0.339	-8.4 (-11.3, -5.4)	1.9 (-2.9, 6.0)	**<0.001**	0.061
			5.2 (4.8, 6.1) at 3 months	**<0.001***			6.0 (5.4, 7.6) at 3 months	0.339	-9.1 (-10.8, -2.7)	2.9 (-4.4, 7.3)	**0.002**	**0.034**
C–reactive protein, CRP (mg/dl)	0.3 (0.1, 0.7)	0.2 (0.1, 0.7)	0.4 (0.2, 0.80)	0.465	0.5 (0.2, 1.0)	0.8 (0.3, 2.2)	0.9 (0.5, 1.7)	0.100	10.9± 70.6	42.5 (-15.1, 143.3)	**0.032**	**0.004§**
Erythrocyte sedimentation rate, ESR (mm/h) †	37 (24, 62)	30 (20, 50)	32 (25, 45)	0.056	50 (41, 73)	55 (27, 62)	33 (27, 50)	0.273	-8.0± 25.6	-34.6 (-60.5, 19.9)	0.123	0.382
Total cholesterol (mg/dl)	163 ± 42	149 ± 33	146±30	**0.020**	164 ± 39	164± 41	162± 38	0.389	-8.4± 16.4	-1.0± 7.1	0.062	0.269
Triglycerides (mg/dl)	222 ± 88	167 ± 69	144 ±66	**<0.001**	188± 71	177 ± 71	180 ± 69	0.337	-30.9± 25.4	-1.6± 25.1	**0.001**	0.469 **0.05****

*Comparisons of baseline with 3 months value.

** P=0.050 for comparisons of triglycerides at 2 months follow-up.

Normally distributed variables are shown as mean± standard deviation. Non-normally distributed variables are shown as median and interquartile range.

†The variables were transformed as follows to achieve normality and conduct parametric tests for comparisons. Reversed variables (1/variable) at baseline and follow-up: glucose, HbA1c. Logarithmized variables (log variable) at baseline and follow-up: Erythrocyte sedimentation rate (ESR)

§ For C-reactive protein the Kruskal-Wallis test was performed, since the normality criterion was not fulfilled.

Supplementary Table S3: Correlations of hematological, biochemical and molecular markers in the control population at follow-up.

		sRAGE	CML	RAGE	COX
sRAGE (pg/ml)	Spearman rho	NA	**-0.967****	**0.497***	0.379
	P-value		**<0.001**	**0.030**	0.110
CML (ng/ml)	Spearman rho	**-0.967****	NA	**-0.494***	-0.357
	P-value	**<0.001**		**0.044**	0.160
RAGE protein expression	Spearman rho	**0.497***	**-0.494***	NA	0.590**
	P-value	**0.030**	**0.044**		0.008
COX protein expression	Spearman rho	0.379	-0.357	**0.590****	NA
	P-value	0.110	0.160	**0.008**	
CRP (mg/dL)	Spearmanrho	-0.365	0.437	-0.388	-0.074
	P-value	0.113	0.069	0.101	0.764
ESR (mm/h)	Spearmanrho	0.215	-0.243	0.281	-0.061
	P-value	0.407	0.365	0.292	0.821

Glucose (mg/dL)	Spearman rho	0.388	-0.443	**0.467***	**0.481***
	P-value	0.091	0.066	**0.044**	**0.037**
Urea (mg/dL)	Spearman rho	0.343	-0.453	**0.461***	0.334
	P-value	0.139	0.059	**0.047**	0.162
Creatinine (mg/dL)	Spearman rho	-0.062	0.080	**0.464***	0.251
	P-value	0.796	0.753	**0.045**	0.299
Uric acid (mg/dL)	Spearman rho	0.236	-0.293	0.262	0.223
	P-value	0.316	0.238	0.278	0.359
GGT (U/L)	Spearman rho	-0.014	0.084	0.253	0.147
	P-value	0.952	0.742	0.297	0.548
Total protein	Spearman rho	0.380	-0.359	**0.465***	**0.519***
	P-value	0.108	0.157	**0.045**	**0.023**
Albumin (mg/dL)	Spearman rho	0.215	-0.185	**0.464***	0.365
	P-value	0.377	0.478	**0.045**	0.125

Cholesterol (mg/dL)	Spearman rho	0.394	**-0.469***	0.000	-0.159
	P-value	0.086	**0.050**	0.999	0.516
Triglycerides (mg/dL)	Spearman rho	0.344	-0.237	0.169	0.235
	P-value	0.137	0.344	0.490	0.332
HDL-cholesterol (mg/dL)	Spearman rho	0.347	-0.422	0.230	-0.102
	P-value	0.134	0.081	0.343	0.678
LDL-cholesterol (mg/dL)	Spearman rho	0.236	-0.365	-0.018	-0.126
	P-value	0.316	0.136	0.943	0.606

Supplementary Table S4: Correlations of hematological, biochemical and molecular markers in the intervention population at follow-up.

		sRAGE	CML	RAGE	COX
sRAGE (pg/ml)	Spearman rho	NA	**-0.957****	0.186	-0.134
	P-value		**<0.001**	0.431	0.573
CML (ng/ml)	Spearman rho	**-0.957****	NA	-0.111	0.113
	P-value	**<0.001**		0.640	0.635
RAGE protein expression	Spearman rho	0.186	-0.111	NA	0.336
	P-value	0.431	0.640		0.147
COX protein expression	Spearman rho	-0.134	0.113	0.336	NA
	P-value	0.573	0.635	0.147	
CRP (mg/dL)	Spearman rho	-0.236	0.246	-0.281	-0.030
	P-value	0.291	0.269	0.230	0.899
ESR (mm/h)	Spearman rho	0.116	-0.069	-0.188	-0.094
	P-value	0.609	0.761	0.427	0.693
Glucose (mg/dL)	Spearman rho	0.132	-0.089	0.405	0.352
	P-value	0.557	0.694	0.076	0.128

Urea (mg/dL)	Spearman rho	-0.062	-0.063	-0.277	0.002
	P-value	0.785	0.780	0.237	0.995
Creatinine	Spearman rho	-0.286	0.275	-0.074	-0.222
	P-value	0.196	0.216	0.758	0.347
Uric acid (mg/dL)	Spearman rho	-0.119	0.013	-0.006	0.024
	P-value	0.599	0.954	0.980	0.919
GGT (U/L)	Spearman rho	0.053	-0.125	-0.319	-0.221
	P-value	0.814	0.581	0.170	0.349
Total protein (mg/dL)	Spearman rho	-0.157	0.079	0.071	0.234
	P-value	0.485	0.728	0.767	0.320
Albumin (mg/dL)	Spearman rho	-0.259	0.221	0.326	0.433
	P-value	0.244	0.322	0.161	0.056

Cholesterol (mg/dL)	Spearman rho	-0.269	0.289	0.430	**0.606****
	P-value	0.225	0.192	0.058	**0.005**
Triglycerides (mg/dL)	Spearman rho	0.322	-0.253	**0.579****	0.332
	P-value	0.144	0.257	**0.007**	0.152
HDL-cholesterol (mg/dL)	Spearman rho	-0.250	0.311	0.174	**0.530***
	P-value	0.261	0.159	0.463	**0.016**
LDL-cholesterol (mg/dL)	Spearman rho	-0.420	**0.436***	0.190	**0.593****
	P-value	0.052	**0.042**	0.423	**0.006**

Supplementary Table S5: Dietary recommendations in the low dietary AGES intervention

GREEK REPUBLIC
UNIVERSITY OF THE PELOPONNESE
FACULTY OF HEALTH SCIENCES
DEPARTMENT OF NURSING

GENERAL INSTRUCTIONS

- If you consume milk, yogurt or cheese, you should choose low-fat products. You should avoid feta or yellow cheese. If you wish to consume cheese, prefer low-fat cheese.
- If you consume crackers or toasted bread, you can replace it with slices of bread that is not baked/ toasted.
- You should avoid processed meats (e.g. ham, bacon).
- You should avoid canned goods.
- You should avoid fried foods (fried meat, fish, potatoes, eggs, sweets such as donuts, pancakes, bagels, donuts, etc.).
- You should avoid grilled foods (grilled meat, fish, vegetables, etc.).
- You should not consume ready-made food (e.g. souvlaki, gyros, pizza, club sandwich, etc.). - You should not consume chips, shrimps, cookies, dry-baked, roasted nuts, crackers, and mayonnaise

- You should avoid alcohol and soft drinks.

Cooking tips

- You may eat boiled meat or roasted meat in the oven (cooked at a maximum of 180° or in the pot on low heat until cooked) - When cooking (meat, fish or vegetables) in the pot don't sauté-tear it (nor the onion)
- You should not use a pressure cooker nor pan.
- When you cook meat, you can marinate it with vinegar or juice from fresh lemons for an hour.
- The oil (mostly olive oil), where possible should be added at the end of cooking (e.g. legumes).
- For legumes, please prefer to put the oil on the plate and not on the pot.
- Replace grilled meats with meats that are baked in the oven at a low temperature

We know it's not easy to change your diet, since you may be under other dietary restrictions due to health problems. However, with your help, we hope we will be able to help patients suffering from Chronic Kidney Disease in the future to:

- **slow down the progression of the disease**
- **reduce long-term complications of the disease**

Together we strive to improve the lives of patients who suffer from Chronic Kidney Disease.

Thank you very much for your help

At your disposal for any information and assistance
